# Metastasis of Gastric Signet-Ring Cell Carcinoma to the Urinary Bladder: A Case Report and Review of the Literature

**DOI:** 10.1155/2015/127516

**Published:** 2015-08-04

**Authors:** Kerem Okutur, Orhan Onder Eren, Gokhan Demir

**Affiliations:** ^1^Department of Medical Oncology, Acibadem University School of Medicine, Buyukdere Cad, No. 40, Sariyer, 34453 Istanbul, Turkey; ^2^Department of Medical Oncology, Yeditepe University School of Medicine, Devlet Yolu, Ankara Caddesi, No. 102-104, Kozyatagi, 34652 Istanbul, Turkey

## Abstract

Although signet-ring cell (SRC) adenocarcinoma is commonly seen in the stomach, it is a very rarely seen histologic entity in the bladder. It is difficult to distinguish primary SRC adenocarcinoma of the bladder from bladder metastasis of SRC carcinoma of the stomach only based on histological findings. In such cases, clinical findings and immunohistochemical studies may be helpful. We present here a 48-year-old male patient presenting with hematuria and abdominal pain. Computerised tomography of the patient revealed a gastric mass, peritoneal involvement, and thickening of the bladder wall, and histopathological analysis revealed SRC adenocarcinoma in both of the endoscopic biopsies taken from the stomach and bladder. Immunohistochemical analyses confirmed the diagnosis of SRC adenocarcinoma of the bladder secondary to gastric cancer.

## 1. Introduction

Ninety-five percent of primary bladder tumors have transitional cell carcinoma histology. Adenocarcinomas of the bladder constitute only 1% of all bladder tumors and usually emerge as a result of metastatic involvement of the bladder. Metastatic bladder tumors are responsible for less than 2% of all bladder tumors and originate most commonly from melanoma, breast cancer, and gastric cancer. Curative surgery is the gold standard in the treatment of primary bladder adenocarcinomas; on the other hand, secondary bladder adenocarcinomas have no chance of cure and chemotherapy or radiotherapy is administered for palliative purposes [1].

Signet-ring cell (SRC) carcinoma is a subtype of mucin producing adenocarcinomas. Ninety percent of SRC tumors arise from stomach, colon, and breast. SRC form is associated with aggressive clinical course and early metastatic disease particularly in tumors of gastrointestinal origin [2, 3]. In bladder tumors, SRC histologic type is very rare. When SRC carcinoma histology is encountered in the bladder of a patient, primary SRC carcinoma of the bladder and bladder metastasis of a malignancy of gastrointestinal system origin are primarily included in the differential diagnosis [4]. It is important to distinguish these two conditions because their treatment and prognosis are different. It is however difficult to differentiate between primary and secondary SRC carcinomas of the bladder both clinically and histologically.

We present here a case presenting with urinary system symptoms and found to have bladder metastasis secondary to SRC of the stomach.

## 2. Case Report

**Figure 1:**
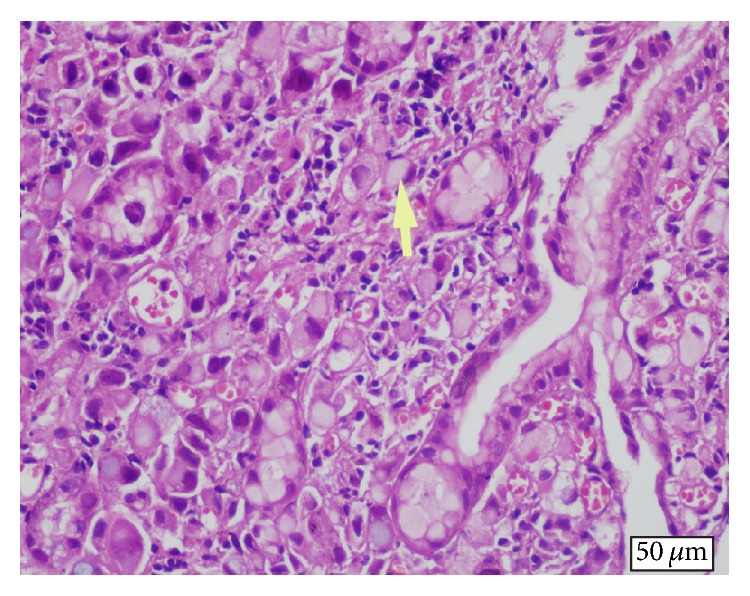
Primary signet-ring cell (arrow) carcinoma of the stomach (H&E, ×20).

**Figure 2:**
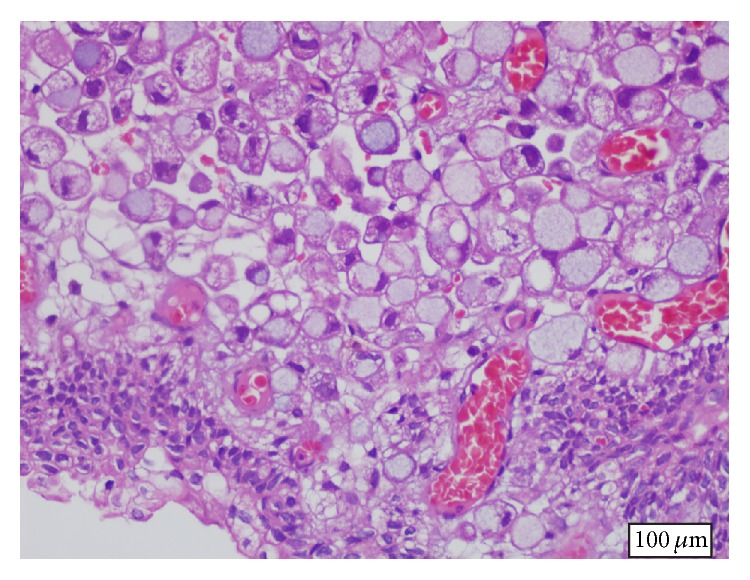
Signet-ring cell carcinoma infiltrating bladder subepithelium (H&E, ×20).

48-year-old male patient presented to the urology clinic with complaints of gross hematuria and abdominal pain of duration of a few weeks. In addition, he also described loss of appetite, weight loss, and fatigue. His ECOG performance status was 1. Physical examination revealed mild abdominal distention and tenderness in the hypogastric region with deep palpation; there was no defence or rebound. Laboratory workup was as follows: hemoglobin 10.5 g/dL, creatinine 1.0 mg/dL, carcinoma antigen (CA) 19.9 168 mg/dL, and carcinoembryonic antigen (CEA) 9.2 mg/dL. Abdominal tomography revealed a malignant tumoral mass in gastric corpus, peritoneal involvement and ascites, multiple abdominal lymphadenopathies, bilateral grade 1 hydronephrosis, and diffuse thickening of the bladder wall. Endoscopy of the upper gastrointestinal system revealed an infiltrating mass of malignant appearance in the gastric corpus. Pathologic examination of endoscopic biopsy material taken from the mass was consistent with SRC carcinoma (Figure 1). In immunohistochemical analyses, cell blocks obtained from mass biopsy and ascites fluid stained positive for CEA and cytokeratin 7 (CK7) and negative for cytokeratin 20 (CK20); in addition, there was focal staining with mucicarmine (MUC). All these findings were suggestive of a gastric primary carcinoma. Papillary-nodular lesions diffusely covering the bladder wall were noted in cystoscopy. Transurethral biopsy was consistent with glandular differentiation and intact urothelial epithelium with SRC carcinoma infiltrating the subepithelium (Figure 2). In immunohistochemical analyses, CEA and CK7 were positive and CK20 was negative, similar to the biopsy taken from the stomach. The patient was started on systemic chemotherapy consisting of docetaxel, cisplatin, and 5-fluorouracil (modified DCF) with the diagnosis of metastatic gastric cancer. A partial response was noted in the radiologic imaging performed after the second cycle. The patient whose hydronephrosis regressed and hematuria did not recur is in the seventh month of his diagnosis and his clinical status is stable.

## 3. Discussion

Most of the information about metastatic tumors of the bladder is derived from autopsy series. When the primary tumor is prostate, colon, rectum, or cervix, bladder is involved with direct extension; on the other hand, in melanomas and breast and gastric cancers, bladder metastases occur as a result of lymphatic/hematogenous spread or peritoneal dissemination [5]. In a series of 282 patients including secondary tumors of the bladder, the tumors most commonly causing bladder involvement with direct extension were colon (21%), prostate (19%), rectum (12%), and cervix (11%) [6]. However, when tumors involving the bladder with metastatic spread are investigated, gastric cancer is the leading cause (4.3%) followed by melanoma (3.9%), lung (2.8%), and breast (2.5%) cancer. In this series, SRC histology is present in only 3 of 12 reported cases of gastric cancer. In our case, the presence of ascites of malignant nature and intra-abdominal metastatic lymph nodes suggests that bladder metastasis may have developed as a result of lymphatic/hematogenous and/or peritoneal dissemination. While the usual metastatic pattern of gastric cancer generally occurs in the form of lymph nodes and peritoneal and liver metastases, gastric cancers with SRC histology have been reported to exhibit a different pattern of metastasis. Peritoneal metastases are more commonly seen in SRC gastric cancers; in addition, pulmonary involvement via lymphatic route, ovarian metastases, and atypical metastases are more common [7].

Including our case, there are 16 cases in the English literature reporting bladder metastasis secondary to gastric cancer [8–19] (Table 1). The majority of the cases are above 50 years of age. Synchronous bladder metastasis was noted during the diagnosis of primary gastric tumor in only 5 cases, and, in the remaining 11 cases, bladder metastasis was noted at a time frame later (median 24 months later, range 7–120 months) than the diagnosis of gastric cancer. While there was isolated bladder metastasis in nine cases, metastatic disease was present at the time of diagnosis in the other 7 cases. Ten of sixteen cases have SRC adenocarcinoma. Metastatic disease was present at the time of diagnosis in 6 of these 10 cases and peritoneal involvement was detected in six cases; however, metastatic disease was encountered at the time of diagnosis in only 1 of 6 cases without SRC histology. This is consistent with the aggressive clinical course of SRC gastric cancers.

In bladder metastases, urinary system findings are present at the time of diagnosis in approximately 20% of the cases [11]. In cases where the tumor is a focal protuberant lesion, macroscopic hematuria is a common sign and this facilitates the diagnosis; on the other hand, symptoms may be more subtle and diagnosis may be more difficult in cases where the bladder wall is diffusely involved. In these cases, irritative symptoms and hydronephrosis are predominant [15]. Hematuria appears to be the most common presenting symptom in cases with bladder metastasis due to gastric cancer reported in the literature. Hydronephrosis was noted in seven cases at the time of presentation and the tumor in the bladder is characterized with diffuse wall thickening in 5 of these 7 cases. Our case presented with macroscopic hematuria and papillary-nodular metastatic lesions diffusely involving the bladder wall were noted in cystoscopy. In addition, there was hydronephrosis associated with wall thickening due to the diffuse involvement of the bladder. Double J stent was not found to be necessary because hydronephrosis was still at an early stage and renal functions and urinary output were normal. Imaging performed after systemic chemotherapy revealed that hydronephrosis disappeared and thickening of the bladder wall regressed.

Histologically, bladder adenocarcinomas represent less than 2% of primary bladder tumors and 54% of secondary tumors [17]. Primary SRC bladder adenocarcinomas are much more rare and account for 0.24% of all bladder malignancies [18, 19]. SRC histology is more common in gastrointestinal system tumors and particularly in gastric cancer and is seen in 3.4% to 39% of the patients [20]. As is true for gastrointestinal tumors, SRC histology is also associated with “unfavorable outcomes” in primary bladder adenocarcinomas [21]. However, long-term survival is possible with radical cystectomy in primary SRC bladder cancers [1]. Since the treatment approaches are very different, it is important to distinguish between primary and secondary bladder adenocarcinoma. Mostofi et al. have reported that polypoid formation or presence of Brunn's nests in the tumor and glandular or mucous metaplasia in the adjacent mucosa and presence of epithelial cell foci such as squamous or transitional cells should suggest primary origin [22]. Still, despite all these clues, it may not be possible to histologically differentiate between primary and secondary adenocarcinomas of the bladder. Immunohistochemical studies may be helpful at this stage. CK7 is positive in 82% and CK20 in 73% of primary bladder tumors, whereas 29% of the cases are CK7 negative and CK20 positive [23]. In addition to immunohistochemical CK7 and CK20 positivity, negative caudal-type homeobox 2 (CDX2) which is frequently expressed in tumors of gastrointestinal origin suggests primary bladder tumor. CK7 negativity, CK20 positivity, and CDX-2 positivity are a frequently seen pattern in gastrointestinal cancers and particularly in colorectal cancer. In gastric cancer, CK7 is usually positive and CK20 is negative. Positive expression rates of CK7 and CK20 in intestinal type of primary gastric cancer are 63% and 32%, respectively, whereas these rates are 75% and 42% in diffuse SRC type [24]. MUC is positive at a high rate in mucin producing tumors and particularly in gastrointestinal malignancies [25]. With their introduction into frequent use, immunohistochemical studies have been used for confirming the diagnosis in nearly all the patients with bladder metastases due to gastric cancer reported in the literature since 2011. Our case also had typical SRC adenocarcinoma histology in both the stomach and bladder tumors. On the other hand, the tumor in the bladder had typical glandular differentiation and urothelial epithelium was intact. While the tumor in the stomach stained positively with CK7, CEA, and MUC, there was no staining with CK20; immunohistochemical staining pattern of the biopsy taken from the bladder was the same as that taken from the stomach and this staining pattern was consistent with primary gastric adenocarcinoma.

Prognosis is poor in bladder metastases of gastric origin. In localized primary SRC carcinoma of the bladder, long-term disease-free survival is possible with radical surgery and adjuvant therapy [1]. However it is not possible to obtain cure with surgery in bladder metastases because of the aggressive biology of SRC gastric cancer and rapid metastatic burden. Chemotherapy and radiotherapy can slow progression of the disease in some cases and control hematuria and irritative symptoms.

In conclusion, bladder metastasis originating from SRC gastric carcinoma is a rarely seen clinical condition with poor prognosis. Bladder metastasis is frequent particularly in cases with gastric cancer presenting with thickening of bladder wall or a mass accompanying peritoneal involvement. Immunohistochemical studies should be used in cases where it is difficult to clinically and histologically distinguish it from primary SRC adenocarcinoma of the bladder because treatment approaches are different.

## Figures and Tables

**Table 1:** Cases of bladder metastasis secondary to gastric cancer in the literature.

Author/year	Number of patients	Age	Gender	Histology/İHC	Interval between primary gastric tumor and bladder metastasis	Distant metastatic sites at the time of diagnosis	Macroscopic features of bladder metastasis	Urinary symptoms	Presence of hydronephrosis	Treatment of bladder metastasis	Prognosis
Saba et al. [8] (1997)	1	52	M	SRCC/no	7 years	Peritoneum, pleura, lymph nodes	Protuberant mass	Hematuria	No	No treatment	Died

Ota et al. [9] (1999)	1	57	F	Adenoca/no	2 years	No distant metastasis	Diffuse wall thickening	Incontinence	Yes	Chemotherapy	Alive after 12-month follow-up

Kim et al. [10] (2001)	3	60	M	Adenoca/no	1 year	No distant metastasis	Protuberant mass	Dysuria, sense of residual urine	NR	NR	NR
		57	F	SRCC/no	15 months	No distant metastasis	Diffuse wall thickening	Dysuria, frequency	NR	NR	NR
		42	M	SRCC/no	2 years	No distant metastasis	Diffuse wall thickening	Dysuria	NR	Total cystectomy	NR

Antunes et al. [11] (2004)	1	63	F	SRCC/no	21 months	Peritoneum	Diffuse wall thickening	Dysuria, lumbar pain	Yes	No treatment	Alive after 8-month follow-up

Matsuhashi et al. [12] (2005)	1	90	F	Adenoca/no	Synchronous metastasis	No distant metastasis	Protuberant mass (in a bladder diverticulum)	Hematuria	No	No treatment	Died 3 months after the diagnosis

Farhat et al. [13] (2007)	1	58	M	Adenoca/no	15 months	No distant metastasis	Protuberant mass	Hematuria	No	TUR	NR

Lim et al. [14] (2011)	1	51	M	Adenoca/no	2 years	Peritoneum, bone	Protuberant mass	Hematuria	Yes	Partial cystectomy	Died 7 months after the surgery

Sharma et al. [15] (2011)	1	30	M	SRCC/no	2 years	No distant metastasis	Protuberant mass	Hematuria	No	TUR, chemotherapy	Alive 5 months after the chemotherapy

Neves et al. [16] (2011)	2	62	F	SRCC/yes	Synchronous metastasis	Peritoneum, ovary (Krukenberg)	Diffuse wall thickening	Dysuria, infrequency, lumbar pain	Yes	No treatment	Died
		41	M	SRCC/no	Synchronous metastasis	No distant metastasis	Protuberant mass	Hematuria	No	Partial cystectomy	Died

András et al. [17] (2013)	1	59	M	Adenoca/yes	10 years	No distant metastasis	Protuberant mass	Hypogastric pain	No	TUR, chemotherapy	NR

Vigliar et al. [18] (2013)	1	38	M	SRCC/yes	7 months	Peritoneum, pleura, lymph nodes	Protuberant mass	Hematuria	Yes	No treatment	Died 2 months after the diagnosis

Kalra et al. [19] (2015)	1	60	M	SRCC/yes	Synchronous metastasis	Peritoneum	Diffuse wall thickening	LUTS	Yes	Chemotherapy	Alive (follow-up NR)

Present case	1	48	M	SRCC/yes	Synchronous metastasis	Peritoneum, lymph nodes	Diffuse wall thickening	Hematuria	Yes	Chemotherapy	Alive 5 months after the diagnosis

M, male; F, female; İHC, immunohistochemical studies; SRCC, signet-ring cell carcinoma; Adenoca, adenocarcinoma; LUTS, lower urinary tract symptoms; TUR, transurethral resection; NR, not reported.
